# Puerperal Insanity

**Published:** 1849-05

**Authors:** 


### OBSTETRICS.

*Puerperal Insanity.* By Dr. Webster.—In a valuable communication to the Westminster Medical Society, on the subject of puerperal insanity, Dr. Webster entered at considerable length into the statistics of the disease. To illustrate its frequency as compared with that of other forms of mental derangement, he stated, that in 1091 curable female patients recently attacked by insanity, and admitted into Bethlehem Hospital, during the last six years, 131, or one-eighth of the whole, were puerperal cases; thus showing that the malady is not so unfrequent as many may perhaps believe. Again as to the curability of this form of mania, more recoveries were reported than in the other varieties of lunacy; 81 puerperal patients having been cured, or at the rate of 61.83 per cent.; whereas the average recoveries during the last twenty years, in all cases of insane females treated at this institution, was 53.67 per hundred. Hence, three in every five cases of puerperal insanity may be confidently expected to get well within a year. In regard to hereditary tendency to mental disease, 51 of the 131 patients were so predisposed, or 30 per cent.; whilst 41 were suicidal, being at the rate of 31 in every 100. Both these peculiarities are of much importance in this malady, and materially influence the disease, its progress, and result. The total deaths in the 133 puerperal patients amounted to six, or four and a half per cent., thus making the average rate of mortality nearly the same as in the other species of insanity, taken collectively. The particulars of the cases, and pathology, next occupied attention, and Dr. Webster stated, that three of the six patients who died were suicidal and hereditary; one was only hereditarily predisposed to insanity, but not suicidal; whilst two, it was reported, had neither of these peculiarities; and none ever were insane previously. In addition to these facts, Dr. Webster also mentioned, that half the deaths occurred in patients who were not affected longer than fifteen days, the shortest period being eleven days, and all were attacked by insanity within seventeen days after their confinement. In none of the dissections were any morbid appearances observed in the abdomen, but the lungs always appeared to be diseased, and also the brain and membranes. The details of one autopsy were then described, as a specimen of the diseased changes of structure frequently met with in puerperal mania, the principal morbid alterations being, turgidity of the blood-vessels of the brain and membrane; large, bloody points on cutting the cerebral substance; slight serous infiltration of the pia mater, and considerable effusion of fluid in the fifth ventricle; adhesion and purulent ulceration were noticed in the left lung, with hepatization in other portions of that organ, and in the right lung partial pneumonia in the congestive stage. Although this patient had been delivered only twenty-six days prior to her death, no corpus luteum could be discovered in either ovary, nor any diseased changes of structure in the abdomen. Notwithstanding it appeared rather a digression, the author, in his paper, remarked, that grangrene of the lungs, however rare an occurrence in persons carried off by bodily disease, but without any mental affection, sloughing of that organ was not unfrequent in lunatics. He said it was so in his own knowledge, and others had also made similar observations, especially in continental asylums for the insane. Dr. Webster afterwards alluded to the treatment of puerperal insanity; and considering cerebral irritation combined with great exhaustion on the nervous system generally, to constitute the true character of this disease, and that it rarely, if ever, proves inflammatory, he thought depletion, or the use of strong antiphlogistic remedies, became very seldom admissible. Leeches appeared in some cases advisable, but even then should be applied with great caution, and their effects carefully watched. As a general maxim, the author advised the same principles to be followed in the treatment of this malady as in delirium tremens, since the nature of the two diseases were somewhat analogous. Opium, camphor, ammonia, and aromatics, with some of the diffusible stimuli, proved excellent remedies, and ought to be chiefly relied upon. When opium fails to procure sleep, so beneficial in this, as, indeed, in every form of insanity—then conium, hyoscyamus, or Indian hemp, may be substituted. Mild purgatives, to open the bowels, and sometimes cathartics should be prescribed; but powerful drastic medicines are seldom advisable. Enemata also are useful, and sometimes with turpentine. When the disease assumes a more chronic form, setons or tissues may be made in the neck, &c. The shower-bath, from its strengthening influence, then acts beneficially, whilst tonic remedies, with more nutritious food, become necessary, and prove advantageous; indeed, low diet is very often prejudicial in insane patients, and it has been long remarked in many asylums, that improved nutriment, especially in lunatics, frequently becomes a powerful means for promoting recovery. In recent cases of puerperal insanity, when the circulation is accelerated, accompanied by evident congestation of the brain, leeches to the temples, and behind the ears, or blisters, might then be applied, and afterwards cooling lotions, with ice to the head; whilst tartar emetic, or ipecacuanha, in nauseating doses, and digitalis, may be administered for the same object. Besides medical treatment, moral means, with judicious occupation and amusements, proper for the patient, must not be overlooked, as these very often constitute aperitive adjuncts in the management of the insane.—*Lancet,* Dec. 2, 1848.

